# Conservation Challenges for Endemic Vascular Plants: Exploring Large‐Scale Patterns in Climate‐Driven Distribution and Distinctive Functional Traits

**DOI:** 10.1002/ece3.71881

**Published:** 2025-07-29

**Authors:** Udayangani Liu, Tiziana Antonella Cossu, Hugh W. Pritchard

**Affiliations:** ^1^ Royal Botanic Gardens, Kew Ardingly UK; ^2^ Kunming Institute of Botany Chinese Academy of Sciences Kunming Yunnan China

**Keywords:** climate‐driven distribution, conservation, endemics, life form, seed dormancy, seed storage behavior

## Abstract

This study addresses global conservation challenges for endemic vascular plants by exploring large‐scale distribution patterns and functional traits influencing their resilience, survival, and regeneration under changing environments. We compiled data for 23,981 endemic vascular plant taxa across 173 ISO countries using publicly available resources. The dataset includes conservation assessments, “Native” occurrences, and key functional traits relevant to ex situ conservation (plant life forms, seed dormancy and seed storage behavior). We analyzed climate‐driven distribution patterns, trait representation, and their relationships with Red List conservation categories. Alarmingly, 58% of endemic vascular plant taxa are designated as extinct or threatened, three times the rate observed in non‐endemics, highlighting a severe conservation crisis. This disproportionate threat reflects their heightened sensitivity to habitat loss, environmental changes, and climate fluctuations. Results indicate that climate‐driven distribution patterns and distinctive plant and seed traits contribute to this trend. Notably, 34% of endemic taxa are “climate specialists”, with 71% already threatened. Extinction risk spans 11 life forms, with over 50% of taxa within these groups currently threatened. Additionally, 91% of endemic taxa exhibit seed dormancy, requiring dormancy‐breaking mechanisms for ex situ conservation. While 82% produce orthodox seeds, suitable for seed banking, this is 10% below the global average. A greater proportion of endemic taxa produce recalcitrant seeds, which cannot be conserved in conventional seed banks and require alternative approaches, such as cryopreservation. Conservation strategies must consider the climate relationships and functional traits that influence plants' resilience. Priority should be given to “climate specialists”, taxa with narrow climate ranges, and those representing susceptible life forms or producing recalcitrant or dormant seeds, which complicate ex situ conservation. Enhancing seed banking strategies to accommodate seed trait variations is essential for long‐term conservation success. Continued research into the storage and propagation of recalcitrant and/or dormant seeds is vital to future reintroduction and recovery efforts.

## Graphical abstract

The study examines conservation challenges for endemic vascular plants globally by analyzing their distribution patterns and traits that influence resilience, survival, and regeneration under changing environmental conditions. Alarmingly, 58% of endemic vascular plant taxa with conservation assessments are designated as extinct or threatened, three times the rate of non‐endemics, highlighting a severe conservation crisis. Our findings indicate that climate‐driven distribution patterns, and unique plant and seed traits contribute to this trend, highlighting conservation strategies must consider the climate relationships and traits that influence plants' resilience and survival in a changing environment.

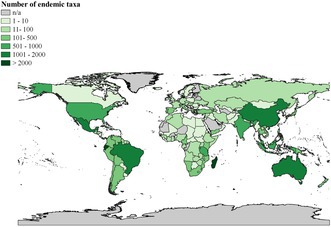

## 1 Introduction

A widely accepted definition of endemism among plants and animals, based on a geographic concept defined by tangible physical boundaries, refers to the ecological state, at any rank (genus, species, subspecies, variety, etc.), of being unique to a defined geographic location, whether large (continent) or small (country, a group of islands, or other defined areas). This uniqueness can arise from historical, ecological, physiological or anthropogenic causes (Major 1988). The geographical boundaries can be natural, administrative or political. Taxa indigenous to a place are termed natives; however, they are not considered endemic if they also occur naturally elsewhere. Their congeners, taxa distributed across a wide geographic range, are termed cosmopolitans or commons. Different perspectives exist regarding the origin of endemics, the processes affecting their populations compared to congeners, and whether they lack common biological traits or possess distinct attributes (Kruckeberg and Rabinowitz 1985). Taxa can originate, exist, and potentially become extinct in the wild. An originally abundant and widespread taxon may eventually become endemic and vice versa. A high degree of endemism is often associated with the age of a clade of common ancestry, geographic isolation, and habitat diversification. These factors influence both the evolution and formation of new endemics (neoendemism) and the survival of relic endemics (paleoendemism) (Major 1988). While neoendemism relates to taxa of recent origin, paleoendemism pertains to ancient relics of taxa that were once more widespread. Classical theories suggest that relics lost their genetic variation through “biotype depletion”, resulting in reduced relict populations that are unable to expand their ranges. Conversely, new endemics may or may not have fully attained their complete distribution ranges (Serrano et al. 2021). Therefore, a country's flora can be characterized by relics and/or new endemics; for example, Uzbekistan's flora contains over 500 relic endemic species (Tojibaev 2010).

The geographic distribution of plant endemism is highly dependent on the taxon (Swenson et al. 2012). Endemic plants are found on all land masses and constitute a significant portion of the world's flora, existing on various scales from continental to regional and local. However, they do not uniformly occupy habitats, exhibiting varying ecological plasticity. Endemics are unevenly distributed across space, along altitudinal gradients, and habitats, with range sizes varying from very narrow to widespread and abundant (Essl et al. 2009; Mráz et al. 2016). Endemics are not necessarily rare: for example, *Pinus sylvestris* (Scots pine), endemic to Eurasia, is locally abundant and dominant in many areas across its extensive geographical range (IUCN 2023). Taxa occurring in small populations in one or a few localities are called restricted‐range or narrow endemics (*sensu stricto*). Steno‐endemics, like *Iris orjenii*, are found in a very limited area and nowhere else in the world.

Knowledge of national‐level endemism for plants, particularly in tropical regions, has been incomplete until the publication of World Checklist of Vascular Plants (WCVP 2020). Approximately 221,399 world's vascular plants are unique or endemic to a single country, with Brazil, Australia and territories, China, South Africa, and Mexico having the highest numbers (Antonelli et al. 2023). Species endemic to a single country represent 46%–62% of global flora and serve as a reasonable proxy for the number of globally threatened plant species within those countries (Pitman and Jørgensen 2002). Sites of global botanical importance (biodiversity hotspots) are identified based on their high level of endemism and species richness. The five leading hotspots with significant plant endemism include the Tropical Andes, Sundaland, the Mediterranean Basin, Madagascar, and Brazil's Atlantic Forest (Myers et al. 2000). Areas with a high degree of endemism tend to have threatened habitats, flora, and fauna (e.g., biodiversity hotspots) as high endemism often correlates with ecological vulnerability and these areas coincide with disproportionate concentrations of ecosystem service values on which local communities depend for their daily livelihoods (Myers et al. 2000; Mittermeier et al. 2011).

Whilst similarities between endemics and cosmopolites exist, endemics tend to exhibit distinct morphological and eco‐physiological traits and preferences. They are often smaller in stature, typically stress‐tolerant, and show altitudinal zonation favoring medium to higher altitudes. Endemics generally inhabit relatively unfertile surfaces, steeper slopes, rocky habitats, and open vegetation. They colonize harsh environments with low competition and disturbance, often featuring smaller flowers with lower investments in pollen transfer and seed production (Médail and Verlaque 1997; Lavergne et al. 2004; van der Werff and Consiglio 2004; Casazza et al. 2005; Broennimann et al. 2006; Alarcón and Cavieres 2018).

Plant diversity faces major threats due to habitat loss, fragmentation and degradation, primarily driven by the intensification of human land use practices, species' overexploitation, competition from invasive species, increased pollution, and anthropogenic climate change (Broennimann et al. 2006; Corlett 2016). About 350,386 species of vascular plants (ferns and allies: pteridophytes and lycophytes; and seed plants: gymnosperms and angiosperms) are known to science, and of these, 45% are at a risk of extinction (Antonelli et al. 2023). The status of endemic plants in Europe drew significant conservation attention, as 50% of its endemic vascular plants were considered in danger of extinction and 64 species were already listed as extinct (Planta Europa 2002). Projections under seven climate change scenarios indicated that over half of 1350 European plant species could be threatened by 2080 (Thuiller et al. 2005). Similar threats have been observed globally. The endemic flora of Southern Africa is predicted to be impacted by a minimum of 41% loss in species richness and a 39% reduction in species distribution range by 2050 due to climate change and land transformation (Broennimann et al. 2006). Southeast Asia, known for its high number of endemics compared to the rest of Asia, ranks third for threatened endemic plant species, with China and Japan occupying the first and second ranks within the region, respectively (ASEAN Centre for Biodiversity 2010). In Türkiye, of the 3504 endemic plant taxa, 12 are estimated to be extinct, with the remaining 99% threatened (Bulut and Yilmaz 2010). In the Mediterranean hotspot of Crete (Greece), paleoendemics have shown a sharper decline in response to bioclimatic and topographic factors, followed by neo‐endemics and then by non‐endemics (Lazarina et al. 2019).

The resilience, survival, and regeneration of plants under changing conditions are not driven by a single factor, but by the dynamic interplay of where they occur, what functional traits they possess, and how well those traits align with environmental conditions (Fernandez‐Pascual et al. 2019). Climate‐driven shifts in plant distribution are a central concern in biogeography and conservation, as they play a pivotal role in determining ecosystem resilience; and in this context, climate specialization, unlike geographically defined endemism, is primarily an ecological concept that is delineated by non‐tangible ecosystem boundaries. More broadly, plant distribution is shaped by complex and interdependent factors, including climatic variables, habitat specificity, and distinctive plant and seed traits. Niche differentiation, primarily influenced by temperature and precipitation, defines the range within which taxa can persist (Alarcón and Cavieres 2018). Generalist plants, occupying a broad niche, may exhibit greater tolerance to climate change than specialists (Broennimann et al. 2006; Joshi and Janarthanam 2004; Alarcón and Cavieres 2018). Conversely, plants restricted to narrow climatic conditions are highly sensitive to climate shifts, resulting in altered distribution, range contraction, habitat loss, or even extinction.

Ex situ conservation, especially seed banking, plays a crucial role in safeguarding plant diversity but faces numerous scientific challenges, spanning from seed collection and processing to long‐term storage and eventual recovery. Challenges are particularly pronounced for “exceptional” plants (*sensu* Pence, Bruns, et al. 2022; Pence, Meyer, et al. 2022), which often exhibit distinctive functional traits that hinder the application of standard banking protocols. Therefore, a deeper understanding of these traits is essential for evidence‐based conservation as they directly influence how taxa respond to environmental stress and how well they perform under ex situ conditions. Such knowledge enables conservation practitioners to identify which taxa are likely to be responsive to seed processing and storage, which may require special interventions, and how to optimize ex situ conservation measures to ensure the long‐term conservation and potential restoration.

Among the functional traits most important for ex situ conservation are plant life forms, as well as seed traits such as dormancy and storage behavior. Life form is a foundational trait that influences how plants fulfill ecological roles, adapt to disturbances, and recover from stress. For example, trees, with long lifespans and slower growth, may take decades to recover after a fire, while herbaceous plants often regenerate rapidly. Dormancy is another critical trait, determined by morphological and physiological properties, preventing seeds from germination, even under environmental conditions that would typically promote germination in non‐dormant (ND) seeds (Simpson 1990). It allows seeds to survive and persist under unfavorable conditions by delaying germination until environmental cues signal a greater chance of seedling survival. Primary dormancy develops during seed maturation on the mother plant (Baskin and Baskin 2004a, 2014). Fresh and mature ND seeds differ categorically from dormant seeds and germinate in various environmental conditions (temperature, photoperiod, precipitation). Seed dormancy is categorized into eight classes (Table 1; Baskin and Baskin 1998, 2004a): four primary types [Physiological (PD), Morphophysiological (MPD), Morphological (MD), and Physical (PY)]; and four other types [Physiophysical (PYPD), Chemical, Mechanical, and DUST]. Storage behavior, which describes seeds' response to dehydration, is categorized into three main classes: orthodox (tolerant), recalcitrant (intolerant) and intermediate (Table 2; Pritchard 2004). Evaluating this trait is critical for seed conservation through seed banking, as conventional techniques involving drying and cooling are suitable only for orthodox seeds, while responses vary among taxa (Liu et al. 2018, 2020, 2023; RBG Kew 2020).

**TABLE 1:** Classes of seed dormancy (Baskin and Baskin 1998, 2004a).

Seed dormancy classes	Description
Physiological (PD)	Physiological inhibition of embryo growth
Morphological (MD)	Characterized by undeveloped embryos at the time of seed dispersal
Morphophysiological (MPD)	A combination of MD + PD
Physical (PY)	Impermeability of seed or fruit coat to water
Physiophysical (PYPD)	A combination of PY + PD
Chemical	Presence of inhibitors in the pericarp
Mechanical	Hard or woody fruit wall
DUST	Small seeds (mostly ≤ 1.0 mm in length) with undifferentiated embryos

*Note:* The primary classes are shaded grey.

**TABLE 2:** Classes of seed storage behavior (Pritchard 2004).

Seed storage behavior classes	Description
Desiccation‐tolerant and long‐lived seeds (orthodox)	Seeds can be dried to low moisture contents (15% equilibrium relative humidity) without damage, often achieving moisture levels much lower than those encountered in nature. They can also be stored at sub‐zero temperatures including −20°C used for conventional seed banking
Desiccation‐sensitive and short‐lived seeds (recalcitrant)	Seeds do not survive significant drying and therefore are unsuitable for long term storage unless cryopreservation can be implemented
Intermediate seeds	Seeds exhibit more desiccation tolerance than recalcitrant seeds but are still less tolerant than orthodox seeds. They generally lose viability more rapidly at seed bank temperature, potentially because of lipid crystallisation (Nadarajan et al. 2023)

Although there is extensive literature on endemic plants covering their distribution, biological, physiological, and evolutionary characteristics, and conservation, much of it focuses on national or regional flora and often lacks trait‐based analysis. Comprehensive, global approaches to endemic flora, especially within a conservation context, remain relatively rare. Despite increasing recognition of the importance of functional traits, many current conservation practices still overlook the critical role that plant and seed traits play. Therefore, the objective of our study is to adopt a global perspective to examine conservation challenges for endemic vascular plants by exploring large‐scale patterns in their climate‐driven distribution and distinctive functional traits.

## 2 Material and Methods

The International Union for the Conservation of Nature's (IUCN) Red List of Threatened Species is widely recognized as the most comprehensive resource for global level conservation assessments regarding the extinction risk of fungi, plants, and animals. We used the IUCN (2020) Red List as our data source, linking conservation assessments to our study. The taxonomic and phytogeographic composition of our sample was shaped by the taxa that had already been evaluated on the IUCN Red List. The IUCN dataset, downloaded on 11 May 2020, included eight separate CSV files and provided global conservation assessments for 39,775 vascular plant taxa at species or infraspecific level. The information included: (1) taxonomy from kingdom to species or infraspecific level, including scientific authorships; (2) name of each geographic locality of taxon assigned with a unique ISO two‐letter country code; (3) origin of taxa within a locality as “Native”, “Introduced”, “Vagrant”, “Reintroduced” or “Origin Uncertain”; (4) IUCN global conservation status across 11 Red List categories, distinguishing taxa as either extinct, threatened (EX, Extinct; EW, Extinct in the Wild; CR, Critically Endangered; EN, Endangered; VU, Vulnerable) or non‐threatened (NT, Near Threatened; LR/nt, Lower Risk/near threatened; LR/cd, Lower Risk/conservation dependent; LR/lc, Lower Risk/least concern; LC, Least Concern; DD, Data Deficient); and (5) plant life or growth form (annuals, succulents, trees, vines, etc.). We strictly adhered to the taxonomic names and nomenclature provided in the IUCN Red List. The only taxonomic reconciliation we conducted was mapping IUCN families to those in WCVP (2020) to ensure consistency.

The individual taxa in the original IUCN data download were not categorized as endemic or non‐endemic. To make this distinction, we processed the IUCN's “countries” CSV file using the following steps: (i) defining endemism as a taxon being “Native” to a single locality, represented by a unique ISO two‐letter country code (hereafter called as ISO country); (ii) excluding locality records where a taxon's origin was not listed as “Native”. This step reduced the dataset to 39,729 taxa; and (iii) for each taxon, counting the number of ISO countries associated with a “Native” origin to identify endemics (count = 1) and non‐endemics (count > 1).

### 2.1 Climate‐Driven Distribution

To explore the bioclimate of the habitat where endemics thrive, we extracted nearly 3 million of their recorded occurrences (taxonomic name, ISO country and geo‐coordinates—latitudes and longitudes) from the Global Biodiversity Information Facility (GBIF 2020, https://www.gbif.org/). This was performed using R software version 4.2.1 (R Core Team 2020) by matching taxonomic names of endemics in our list to those in the GBIF. Scientific authorships of taxonomic names were excluded during name matching due to nomenclature inconsistencies between the IUCN and GBIF. We assumed that any impact from excluding those could be mitigated by maximizing the number of extracted occurrences. The dataset was then refined using MS Access and Quantum GIS software version 3.34.0 (QGIS.org 2020, https://qgis.org/en/site/) by removing records with zero, null or invalid coordinates (e.g., non‐numeric, incomplete, located in the ocean far from land), as well as those occurring outside the taxon's native ISO country. This data cleaning process yielded 2,342,598 occurrences for 16,204 endemic taxa on our list.

Coordinates of these records were then mapped to the zones defined in the Köppen–Geiger climate classification (Köppen 1936) using 1‐km resolution maps described in Beck et al. (2018). As some coordinates did not fall within any zone, only 2,245,515 occurrences representing 16,038 taxa (15,776 species) were retained for the climate‐based analysis. The classification system consists of 30 zones, each designated by a two‐ or three‐letter code that indicates climate type. The system divides the world's terrestrial climate into five major groups, represented by the first letter (Table 3). Each group is further subdivided into zones (Table 4) based on seasonal patterns of rainfall (the second letter) and temperature (third letter). We chose this classification as it provides a well‐established, categorical representation of climate zones that integrates temperature and precipitation and reflects meaningful conditions relevant to plant distribution. It offers a practical and ecologically intuitive framework for summarizing bioclimatic conditions across large datasets such as ours, reduces computational complexity, and ensures ecological interpretability, making it more efficient and less error‐prone than processing raw climate data for each individual coordinate.

**TABLE 3:** Major climate groups in Köppen–Geiger climate classification (Köppen 1936).

Climate group	Letter code	Number of zones	Location
Tropical	A	3	On either side of the equator
Arid/Dry	B	4	Immediately north and south of tropical climates
Temperate	C	9	North and south of the arid/dry climates often overlap in latitude with continental climates in North America and Asia
Continental	D	12	Mid‐latitudes within large landmasses, which are less influenced by oceans
Polar/Alpine	E	2	In the Arctic and Antarctic regions. Alpine (mountain or highland) climate, which includes elevations above the tree line, is described alongside the polar climate
	Total	30	

**TABLE 4:** 30 Köppen–Geiger climate zones (Köppen 1936; Beck et al. 2018).

Zone	Main climate type	Rainfall (precipitation)	Temperature (heat)	Description of climate
*Af*	Tropical (*A*)	Rainforest (*f*)		Rainforest
*Am*	Tropical (*A*)	Monsoon (*m*)		Monsoon
*Aw*	Tropical (*A*)	Savannah (w)		Savanna
*Bsh*	Arid/Dry (*B*)	Steppe (*s*)	Hot (*h*)	Hot semi‐arid
*Bsk*	Arid/Dry (*B*)	Steppe (*s*)	Cold (*k*)	Cold semi‐arid
*Bwh*	Arid/Dry (*B*)	Desert (*w*)	Hot (*h*)	Hot desert
*Bwk*	Arid/Dry (*B*)	Desert (*w*)	Cold (*k*)	Cold desert
*Cfa*	Temperate (*C*)	No dry season (*f*)	Hot summer (*a*)	Humid subtropical
*Cfb*	Temperate (*C*)	No dry season (*f*)	Warm summer (*b*)	Oceanic/subtropical highland
*Cfc*	Temperate (*C*)	No dry season (*f*)	Cold summer (*c*)	Subpolar oceanic
*Csa*	Temperate (*C*)	Dry summer (*s*)	Hot summer (*a*)	Hot‐summer Mediterranean
*Csb*	Temperate (*C*)	Dry summer (*s*)	Warm summer (*b*)	Warm‐summer Mediterranean
*Csc*	Temperate (*C*)	Dry summer (*s*)	Cold summer (*c*)	Cold‐summer Mediterranean
*Cwa*	Temperate (*C*)	Dry winter (*w*)	Hot summer (*a*)	Monsoon‐influenced humid subtropical
*Cwb*	Temperate (*C*)	Dry winter (*w*)	Warm summer (*b*)	Subtropical highland/Monsoon‐influenced temperate oceanic
*Cwc*	Temperate (*C*)	Dry winter (*w*)	Cold summer (*c*)	Cold subtropical highland/Monsoon‐influenced subpolar oceanic
*Dfa*	Continental (*D*)	No dry season (*f*)	Hot summer (*a*)	Hot‐summer humid continental
*Dfb*	Continental (*D*)	No dry season (*f*)	Warm summer (*b*)	Warm‐summer humid continental
*Dfc*	Continental (*D*)	No dry season (*f*)	Cold summer (*c*)	Subarctic
*Dfd*	Continental (*D*)	No dry season (*f*)	Very cold winter (*d*)	Extremely cold subarctic
*Dsa*	Continental (*D*)	Dry summer (*s*)	Hot summer (*a*)	Mediterranean‐influenced hot‐summer humid continental
*Dsb*	Continental (*D*)	Dry summer (*s*)	Warm summer (*b*)	Mediterranean‐influenced warm‐summer humid continental
*Dsc*	Continental (*D*)	Dry summer (*s*)	Cold summer (*c*)	Mediterranean‐influenced subarctic
*Dsd*	Continental (*D*)	Dry summer (*s*)	Very cold winter (*d*)	Mediterranean‐influenced extremely cold subarctic
*Dwa*	Continental (*D*)	Dry winter (*w*)	Hot summer (*a*)	Monsoon‐influenced hot‐summer humid continental
*Dwb*	Continental (*D*)	Dry winter (*w*)	Warm summer (*b*)	Monsoon‐influenced warm‐summer humid continental
*Dwc*	Continental (*D*)	Dry winter (*w*)	Cold summer (*c*)	Monsoon‐influenced subarctic
*Dwd*	Continental (*D*)	Dry winter (*w*)	Very cold winter (*d*)	Monsoon‐influenced extremely cold subarctic
*EF*	Polar/Alpine (*E*)	Frost/ice cap (*F*)		Ice cap
*ET*	Polar/Alpine (*E*)	Tundra (*T*)		Tundra

### 2.2 Distinctive Functional Traits

To examine the representation of traits among endemics, we first compiled the following data: (i) life forms for all endemics, based on categories provided by IUCN (2020); (ii) seed dormancy types at the species, genus, or family level, using information from Baskin and Baskin (2014) and Willis et al. (2014). This resulted in 27,067 seed dormancy records, which were later summarized for 17,332 endemic taxa; and (iii) seed storage behavior for 22,360 endemic taxa, predicted using the tool developed by Wyse et al. (2018b), which estimates seed's desiccation response along a probability gradient from orthodox (0) to recalcitrant (1). Predicted values < 0.5 indicate orthodox seeds, while values ≥ 0.5 suggest recalcitrant seeds. An intermediate class was not distinguished in our analysis, as this requires time course analysis of viability during storage at −20°C (Pritchard 2004). Predictions were based on species‐specific habitat information and a set of traits (e.g., seed weight, woodiness), along with desiccation responses observed in closely related taxa within the same genus, family or order. For some taxa, predictions were not possible due to limitations of the model.

### 2.3 Data Analysis

While we acknowledge that a deeper investigation into the drivers of endemism would benefit from formal hypothesis testing and advanced statistical modeling, such analyses were beyond the scope of our study. Instead, we focused on identifying large‐scale patterns that are often obscured at finer scales but are crucial for understanding global biodiversity distribution. Nevertheless, our findings lay a valuable foundation to inform and guide future hypothesis‐driven research and targeted conservation strategies.

Where possible, comparisons were made between endemics and non‐endemics. Large‐scale patterns were described using descriptive statistics, including total counts, percentages, or variable rankings to minimize the reliance on complex analysis while still effectively capturing and illustrating key trends. Extinct, threatened, and non‐threatened taxa were estimated separately for endemics and non‐endemics. Analysis of native distribution across climate zones and prevalence of distinctive functional traits was conducted exclusively for endemics. We described ecological extremes (“specialists” vs. “generalists”, “endemics” vs. “cosmopolites”), rare, less common, moderate or common categories based on natural breaks in the distribution of data.

Whilst endemism is defined geographically, to highlight ecological extremes of their habitats, we categorized taxa occurring in a single climate zone as “climate specialists”, indicating potentially limited resilience to climate change. Those found in 10 or more zones were categorized as “climate generalist”, reflecting presumed broader ecological adaptability and resilience to climate change. To provide a more nuanced understanding of climate breadth, taxa occurring in two to four zones were considered to have a narrow climate range, while those in five to nine zones were considered to have a moderate climate range. These thresholds were selected to reflect natural breaks in the distribution of climate breadth across taxa and to facilitate meaningful comparisons. We explored whether there is a relationship between climate range and the extinction risk by estimating the Pearson correlation coefficient between the percentage of threatened endemics and the number of different climate zones they inhabit, using R software version 4.2.1 (R Core Team 2020).

We also investigated how functional traits are distributed across plant families, climates, IUCN Red List categories, and/or in relation to other traits considered in the study. Additionally, using the predicted values of seed storage behavior, we assessed the proportion of endemics potentially amenable to conservation through seed banking, particularly across threat categories and among “climate specialists”. Although ferns and allies were excluded, their spores exhibit a similar range of desiccation responses to seeds (Ballesteros et al. 2017).

## 3 Results

**FIGURE 1:**
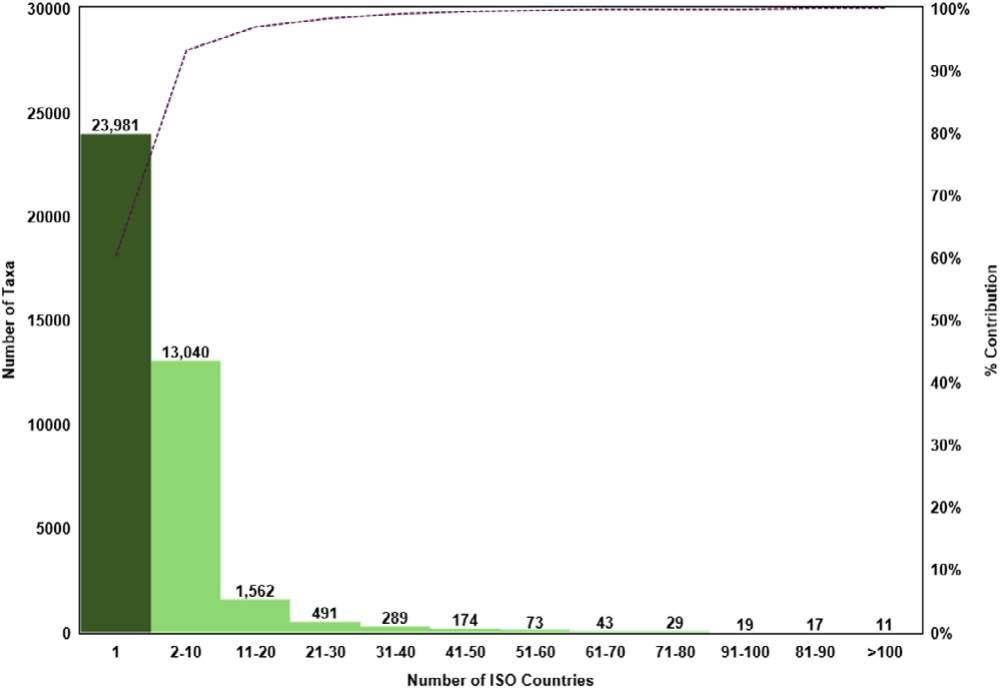
The native distribution range of vascular plants (*n* = 39,729 taxa across 250 ISO countries). Includes both endemics (restricted to a single ISO country) and non‐endemics (grouped into range size‐classes based on the number of ISO countries in which they occur). The *x*‐axis shows range size classes, and the dotted line indicates the cumulative percentage of contribution.

The IUCN (2020) dataset, comprising 39,729 vascular plant taxa, represents the “Native” distribution ranges of global plant diversity, collectively spanning 250 ISO countries. Their “Native” distribution ranged widely, from single ISO country endemics to extremely cosmopolitan species such as *Phragmites australis* , which is native to 244 ISO countries across all nine continents (Figure 1). Notably, only 192 taxa (0.48%) were cosmopolitan, having a native range spanning over 50 ISO countries representing at least two continents. We identified 23,981 endemic taxa (i.e., 60% of the list) across 313 families, 3755 genera, and 23,456 species, each with a native range restricted to a single ISO country. This coverage included 415 ferns and allies and 23,566 seed plants, comprising 723 gymnosperms and 22,843 angiosperms (18,985 dicotyledons + 3858 monocotyledons). We identified eight families rich in endemism, each contributing > 100 genera and/or > 900 taxa (Appendix S1): Fabaceae, Asteraceae, Orchidaceae, Rubiaceae, Poaceae, Cactaceae, Arecaceae, and Myrtaceae. Additionally, there were 21 genera rich in endemism, each contributing > 100 to 717 endemic species (Appendix S2): top five, *Eucalyptus*, *Euphorbia*, *Magnolia*, *Eugenia* and *Sorbus*.

**FIGURE 2:**
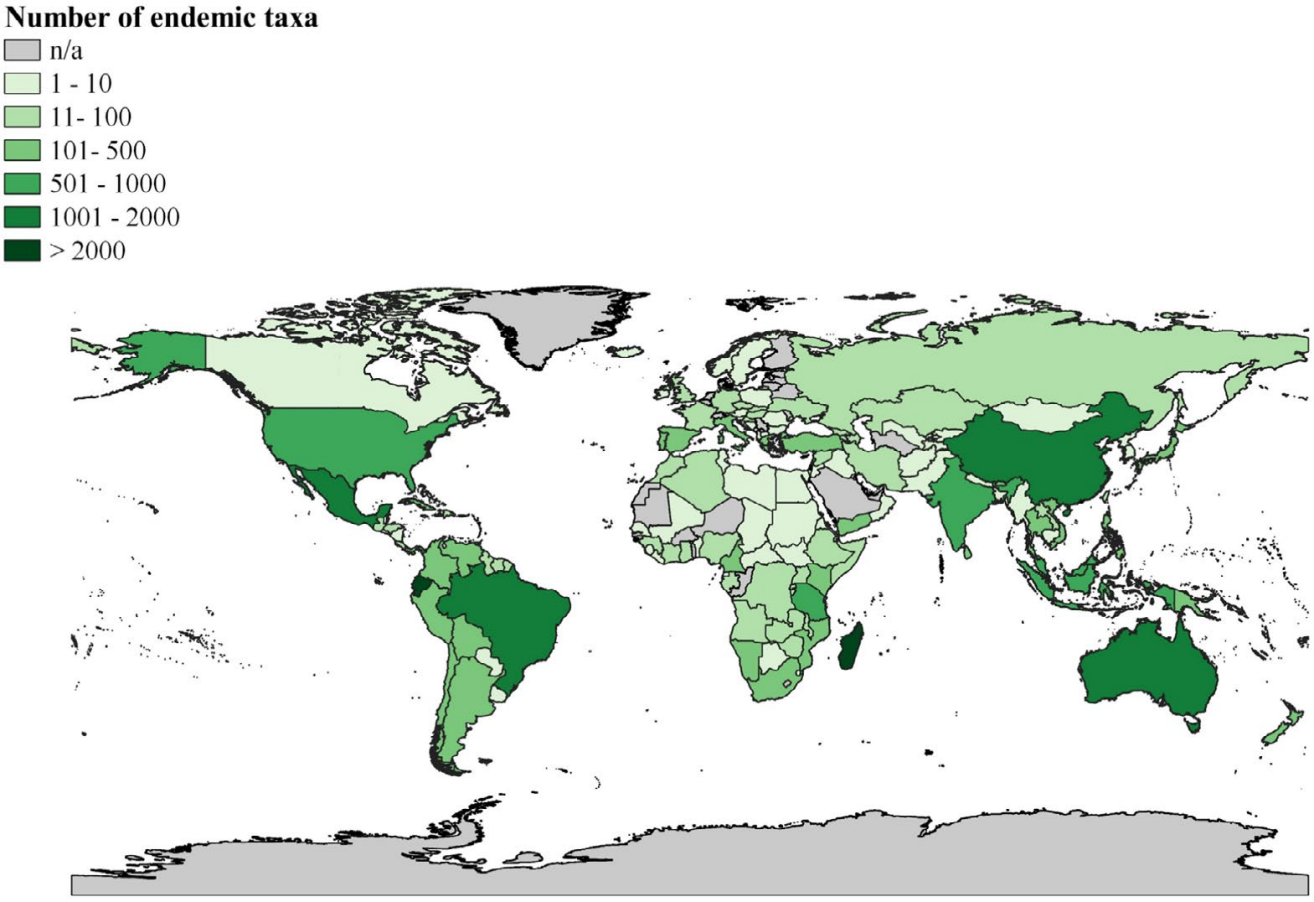
Phytogeographic distribution of endemic vascular plants (*n* = 23,981 taxa across 173 ISO countries).

Endemics originated from all nine continents, spanning 173 ISO countries (Figure 2; Appendix S3), with taxon counts ranging from one (in 20 ISO countries) to 2702. A total of 86 ISO countries had fewer than the median number of taxa (mean = 139; median = 18; standard deviation = 368). When ISO countries were ranked by endemics richness (rank 1 holds the highest taxa count), there were 87 distinct ranks due to ties. The six leading ISO countries, representing five different continents, collectively accounted for 45% of all endemics: Madagascar (Africa); Ecuador (Southern America); Australia (Australasia); Brazil (Southern America); Mexico (Northern America); and China (Asia‐Temperate). The leading contributors from the remaining continents were Malaysia (ranked 7th, Asia‐Tropical), New Caledonia (ranked 9th, the Pacific), Spain (ranked 17th, Europe), and the Falkland Islands: Malvinas (ranked 75th or 13th last, the Antarctic).

**FIGURE 3:**
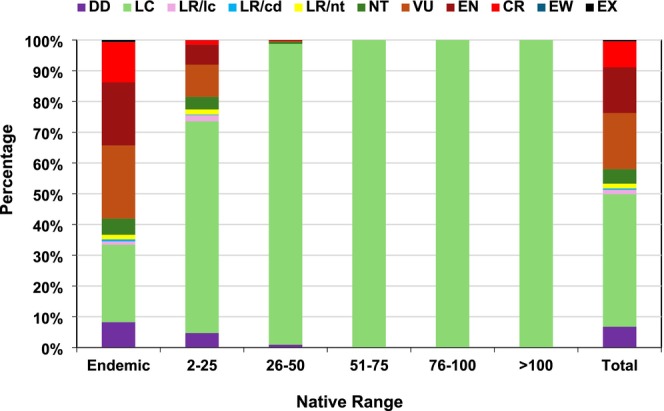
Distribution of IUCN (2020) global Red List categories among vascular plants (*n* = 39,729 taxa across 250 ISO countries). Includes both endemics (restricted to a single ISO country) and non‐endemics (grouped into range size‐classes based on the number of ISO countries in which they occur).

About 58% of endemics (13,922, ~3 times above non‐endemics) were identified in IUCN (2020) as extinct or globally threatened (128 EX, 30 EW, 3144 CR, 4922 EN, 5698 VU) and included 60% of ferns and allies, 52% of gymnosperms, and 58% of angiosperms (Figure 3).

### 3.1 Climate‐Driven Distribution

Among the 16,038 endemic taxa evaluated, diversity was heavily concentrated in tropical (10,325 taxa) and temperate (9888) climates. The Arid/Dry climate (4463) supported moderate diversity, while Polar/Alpine (1331) and Continental (932) hosted the fewest. Endemics were recorded across 27 of 30 climate zones, but their distribution was highly uneven (Table 5). Notably, no endemics were found in three continental zones characterized by extremely cold subarctic climates: *Dfd* (Extremely cold subarctic), *Dsd* (Mediterranean‐influenced extremely cold subarctic) and *Dwd* (Monsoon‐influenced extremely cold subarctic). In the remaining zones, taxa counts ranged from only eight in *Dsa* (Continental, Mediterranean‐influenced hot‐summer humid continental) to over 6000 in *Af* (Tropical, rainforest). The three most taxon‐rich zones were *Af*, *Aw* (Tropical, savannah), and *Cfb* (Temperate, oceanic/subtropical highland), each containing approximately 12%–14% of taxa. Some temperate zones, specifically *Cfc* (Subpolar oceanic), *Csc* (cold‐summer Mediterranean), and *Cwc* (cold subtropical highland/Monsoon‐influenced subpolar oceanic) contained notably fewer endemic taxa than other temperate zones. Among polar/alpine climates, the Ice cap (EF) had far fewer taxa than the Tundra (ET) zone.

**TABLE 5:** Occurrence of 16,038 endemic vascular plant taxa across 30 Köppen–Geiger climate zones.

Zone	Main climate type	Rainfall (precipitation)	Temperature (heat)	Description of climate	Occurrence of endemic taxa^a^	
					Total	%
***Af***	**Tropical (*A*)**	**Rainforest (*f*)**		**Rainforest**	**6098**	**13.80**
*Am*	Tropical (*A*)	Monsoon (*m*)		Monsoon	3560	8.10
***Aw***	**Tropical (*A*)**	**Savannah (*w*)**		**Savanna**	**5956**	**13.50**
*Bsh*	Arid/Dry (*B*)	Steppe (s)	Hot (*h*)	Hot semi‐arid	2841	6.40
*Bsk*	Arid/Dry (*B*)	Steppe (*s*)	Cold (*k*)	Cold semi‐arid	2087	4.70
*Bwh*	Arid/Dry (*B*)	Desert (*w*)	Hot (*h*)	Hot desert	1417	3.20
*Bwk*	Arid/Dry (*B*)	Desert (*w*)	Cold (*k*)	Cold desert	771	1.70
*Cfa*	Temperate (*C*)	No dry season (*f*)	Hot summer (*a*)	Humid subtropical	3615	8.20
***Cfb***	**Temperate (*C*)**	**No dry season (*f*)**	**Warm summer (*b*)**	**Oceanic/subtropical highland**	**5216**	**11.80**
*Cfc*	Temperate (*C*)	No dry season (*f*)	Cold summer (*c*)	Subpolar oceanic	141	0.30
*Csa*	Temperate (*C*)	Dry summer (*s*)	Hot summer (*a*)	Hot‐summer Mediterranean	992	2.30
*Csb*	Temperate (*C*)	Dry summer (*s*)	Warm summer (*b*)	Warm‐summer Mediterranean	1596	3.60
*Csc*	Temperate (*C*)	Dry summer (*s*)	Cold summer (*c*)	Cold‐summer Mediterranean	47	0.10
*Cwa*	Temperate (*C*)	Dry winter (*w*)	Hot summer (*a*)	Monsoon‐influenced humid subtropical	3117	7.10
*Cwb*	Temperate (*C*)	Dry winter (*w*)	Warm summer (*b*)	Subtropical highland/Monsoon‐influenced temperate oceanic	3534	8.00
*Cwc*	Temperate (*C*)	Dry winter (*w*)	Cold summer (*c*)	Cold subtropical highland/Monsoon‐influenced subpolar oceanic	16	0.00
*Dfa*	Continental (*D*)	No dry season (*f*)	Hot summer (*a*)	Hot‐summer humid continental	244	0.60
*Dfb*	Continental (*D*)	No dry season (*f*)	Warm summer (*b*)	Warm‐summer humid continental	304	0.70
*Dfc*	Continental (*D*)	No dry season (*f*)	Cold summer (*c*)	Subarctic	198	0.40
*Dfd*	Continental (*D*)	No dry season (*f*)	Very cold winter (*d*)	Extremely cold subarctic	0	0.00
*Dsa*	Continental (*D*)	Dry summer (*s*)	Hot summer (*a*)	Mediterranean‐influenced hot‐summer humid continental	8	0.02
*Dsb*	Continental (*D*)	Dry summer (*s*)	Warm summer (*b*)	Mediterranean‐influenced warm‐summer humid continental	166	0.38
*Dsc*	Continental (*D*)	Dry summer (*s*)	Cold summer (*c*)	Mediterranean‐influenced subarctic	115	0.26
*Dsd*	Continental (*D*)	Dry summer (*s*)	Very cold winter (*d*)	Mediterranean‐influenced extremely cold subarctic	0	0.00
*Dwa*	Continental (*D*)	Dry winter (*w*)	Hot summer (*a*)	Monsoon‐influenced hot‐summer humid continental	183	0.42
*Dwb*	Continental (*D*)	Dry winter (*w*)	Warm summer (*b*)	Monsoon‐influenced warm‐summer humid continental	347	0.79
*Dwc*	Continental (*D*)	Dry winter (*w*)	Cold summer (*c*)	Monsoon‐influenced subarctic	157	0.36
*Dwd*	Continental (*D*)	Dry winter (*w*)	Very cold winter (*d*)	Monsoon‐influenced extremely cold subarctic	0	0.00
*EF*	Polar/Alpine (*E*)	Frost/ice cap (*F*)		Ice cap	26	0.10
*ET*	Polar/Alpine (*E*)	Tundra (*T*)		Tundra	1325	3.00
				Total number of unique taxa^a^	16,038	

*Note:* The top three zones richest in endemism are shown in bold text; zones with many taxa (> 1000) are in green, those with few taxa (< 100) are shaded in amber, and those with no taxa in grey.

^a^ Some taxa are distributed across multiple zones.

**FIGURE 4:**
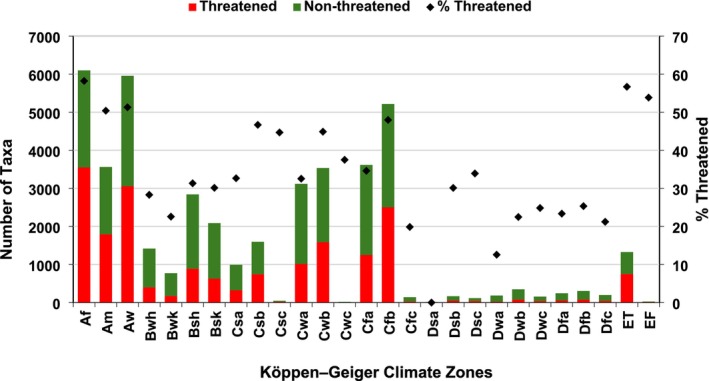
Threatened and non‐threatened endemic vascular plants across climate zones (*n* = 16,038 taxa). Global Red List categories follow IUCN (2020). See Table 4 for climate zone abbreviations.

**FIGURE 5:**
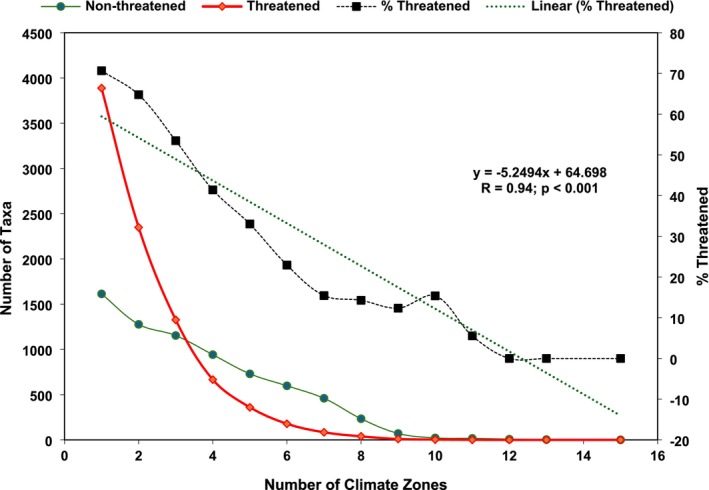
The number of climate zones occupied by threatened and non‐threatened endemic vascular plants (*n* = 16,038 taxa).

A substantial portion of endemics (34%, 5502 taxa) were identified as “climate specialists”, occurring exclusively within a single zone. Only 0.36% of taxa were “climate generalists”, inhabiting 10 or more zones. Nearly half of the taxa (48%) had narrow climate range, occupying two to four zones, while 17% had a moderate climate range, occurring in five to nine zones. “Climate specialists” were found in 25 different zones and included 238 families and 1586 genera, comprising 51 ferns and allies, 138 gymnosperms, and 5313 angiosperms. The three zones with the most “climate specialists” were tropical (*Af*, *Aw*) and temperate (*Cfb*). Only one zone, *Dsa*, hosted no threatened endemics, likely due to its low endemic richness overall (Figure 4). Approximately 50% to 58% of taxa were threatened in tropical climates (*Af*, the highest), with similar percentages in polar/alpine climates, followed by 23% to 31% in arid/dry, 20% to 48% in temperate, and 13% to 34% in continental. About 71% of all “climate specialists” were threatened, with the highest proportion found in tropical climates (64% of taxa), followed by temperate (25%), arid/dry (8%), polar/alpine (2%) and continental (1%). Climate specialization was strongly associated with extinction risk. The threatened taxa decreased as the number of climate zones they occupied increased. This pattern was statistically robust, with a Pearson correlation coefficient of 0.94 (*p* < 0.001) between the number of zones occupied and the percentage of threatened taxa (Figure 5). This strong linear relationship suggests that “climate generalists” are substantially less sensitive to extinction than “climate specialists”.

### 3.2 Distinctive Functional Traits

#### 3.2.1 Life Forms

**FIGURE 6:**
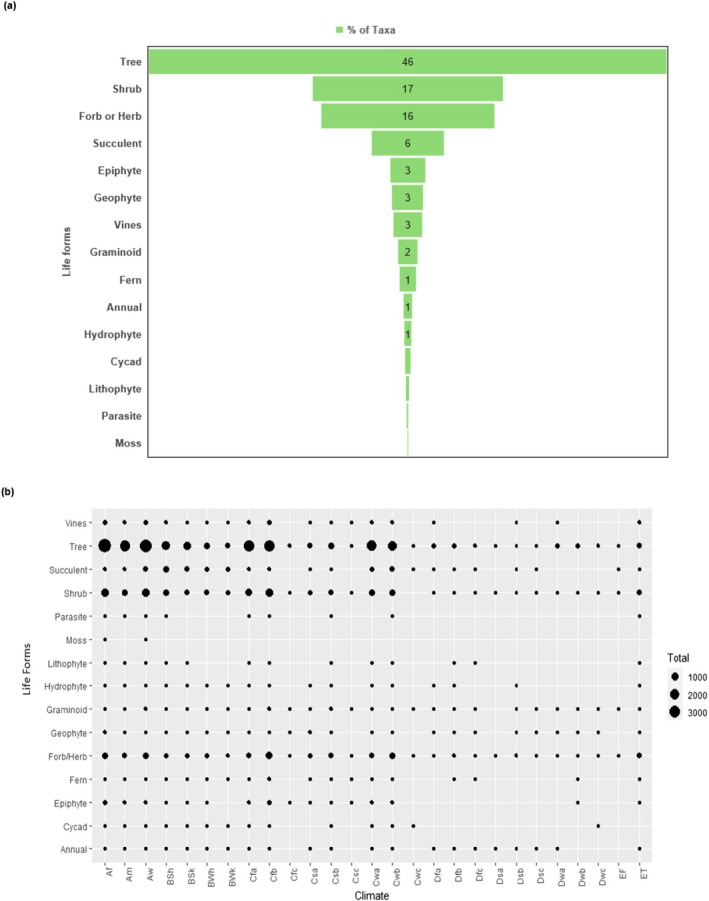
Prevalence of life forms among endemic vascular plants (*n* = 23,981 taxa): (a) overall distribution; and (b) distribution across climate zones. See Table 4 for climate zone abbreviations.

Endemics were distributed across 15 life forms (13 forms for seed plants), with trees comprising the largest proportion (46% of taxa), followed by shrubs (17%) and forbs/herbs (16%) (Figure 6a). Succulents accounted for 6% (primarily as shrubs and trees), while less common forms (epiphytes, geophytes, vines, graminoids and ferns) collectively represented ~12%. Uncommon forms included mosses, parasitic plants, lithophytes, cycads, hydrophytes and annuals.

Endemic trees, shrubs and forbs/herbs occurred across 26 to 27 climate zones; however, their taxon numbers varied substantially across climates. Trees in particular were dominant in eight zones—including all three tropical, four temperate and one arid/dry—where their taxon counts ranged from 1371 to 3924 (Figure 6b). Among the less common or uncommon life forms, graminoids, geophytes and annuals were more evenly distributed, while parasitic plants and mosses appeared more sporadic.

**FIGURE 7:**
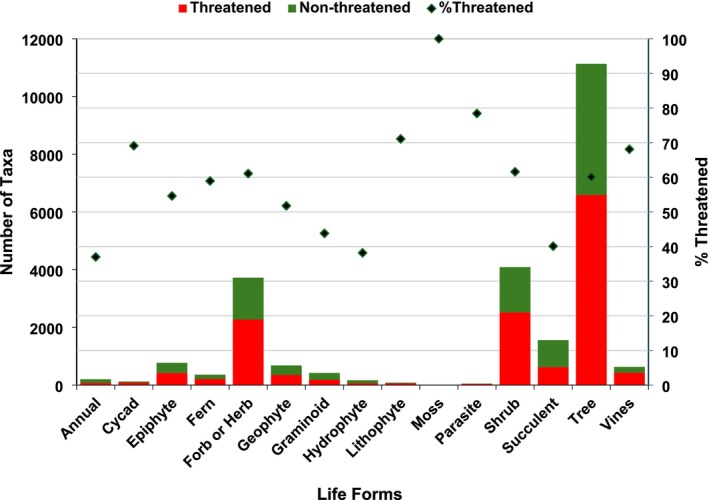
Threatened and non‐threatened endemic vascular plants across life forms (*n* = 23,981 taxa). Global Red List categories follow IUCN (2020).

Eleven life forms had over 50% of their taxa designated as threatened (Figure 7): mosses, parasitic plants, and lithophytes had the highest (> 70%), followed by cycads, vines, shrubs, forbs/herbs, and trees (60% to 69%). Moderate percentages (52% to 59%) were found in ferns, epiphytes, and geophytes, while annuals, hydrophytes, succulents, and graminoids had lower levels (≤ 44%).

#### 3.2.2 Seed Dormancy

**FIGURE 8:**
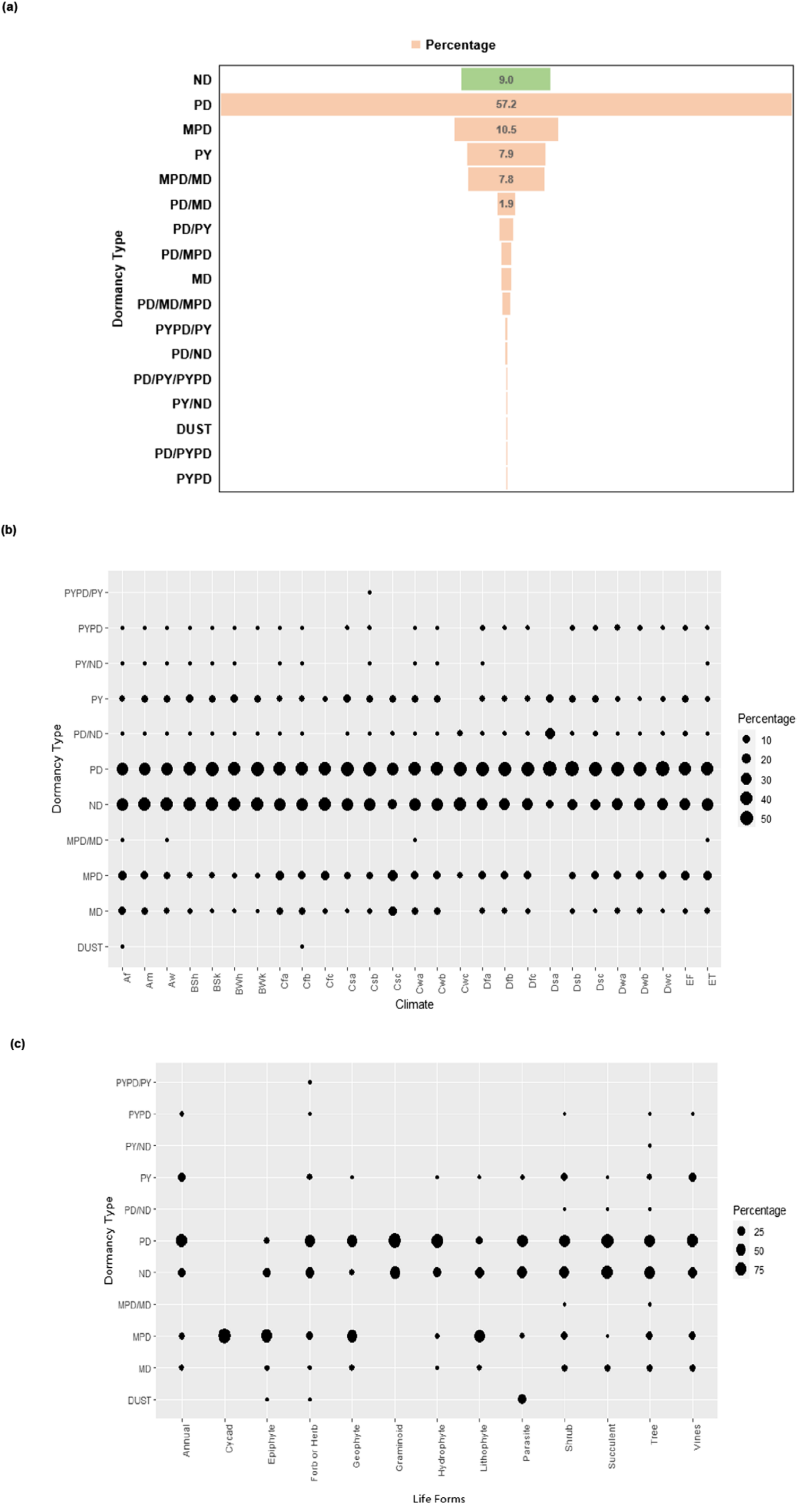
Prevalence of seed dormancy among endemic vascular plants (*n* = 17,332 taxa): (a) overall distribution; (b) distribution across climate zones; and (c) distribution across life forms. Dormancy classes are physiological (PD), morphological (MD), morphophysiological (MPD), physical (PY), and physiophysical (PYPD), and DUST. Taxa producing exclusively non‐dormant (ND) seeds are not differentiated in graph c. See Table 4 for climate zone abbreviations.

Seed dormancy classes examined for 17,332 endemic taxa (16,903 species, 1771 genera, 256 families) included 607 gymnosperms and 16,725 angiosperms (3160 monocotyledons + 13,565 dicotyledons). Importantly, 91% of these taxa produce dormant seeds (Figure 8a), while only 9% have exclusively ND seeds, comprising 29 gymnosperms and 1535 angiosperms (216 monocotyledons + 1319 dicotyledons). Notably, 77% of taxa exhibited a single dormancy, with PD alone being the most common, found in 57% of taxa. Other single‐class dormancy included MPD (~11%), PY (~8%), MD (~1%), and PYPD and DUST (0.1% each). PY and PYPD were exclusive to dicotyledons. About 14% of taxa displayed combinations of multiple dormancies, most commonly the MPD/MD (~8% of taxa), which appear more frequently than MD alone.

All four primary dormancies (PD, MPD, MD, and PY) were distributed across climate zones (Figure 8b), and dormant seeds consistently outnumbered ND seeds. However, no clear climate pattern emerged in the distribution of dormancy. Among 3926 “climate specialists” taxa, only 367 (9%) had exclusively ND seeds.

Dormancy pattern varied notably across life forms (Figure 8c). Taxa with exclusively ND seeds were uncommon across most life forms: none in cycads; 0.36% to < 5% in geophytes, annuals, parasitic plants, graminoids, and vines; 8% to 9% in shrubs, forbs/herbs, and trees; and 13% to 21% in epiphytes, hydrophytes, succulents, and lithophytes. All four primary dormancies were present among nine life forms, with some exceptions: cycads had only MPD, which also dominated in epiphytes, lithophytes, and geophytes; graminoids had only PD, which also dominated in nine other life forms; epiphytes lacked PY; and parasitic plants lacked MD. Infrequent dormancy classes like PYPD appeared among both woody and non‐woody endemics, while DUST was found in parasitic plants, forbs/herbs, and epiphytes.

No clear seed dormancy patterns emerged distinguishing threatened and non‐threatened endemics, suggesting that this trait may be influenced by more complex ecological or evolutionary factors.

#### 3.2.3 Seed Storage Behavior

Seed storage behavior predicted for 22,360 endemic taxa (21,876 species, 3391 genera, 199 families) included 548 gymnosperms and 21,812 angiosperms (3581 monocotyledons + 18,231 dicotyledons). Approximately 82% (18,414 taxa) were predicted to have orthodox seeds. As the intermediate seed storage behavior could not be reliably differentiated, we identified 151 families containing taxa likely to possess orthodox seeds, with no taxa from these families predicted to exhibit recalcitrant storage behavior (Appendix S4). Among these, where at least 100 taxa had predicted storage behavior, 29 families were predicted to be orthodox‐rich (top five with many taxa): Cactaceae, Asteraceae, Orchidaceae, Melastomataceae, and Zingiberaceae. The Annonaceae family showed similar percentages of orthodox and recalcitrant taxa, indicating a more balanced distribution of seed storage behavior; and a further 15 families highlighted a higher prevalence of orthodox taxa (≥ 70%–< 100%) than recalcitrant (top five): Malvaceae, Rosaceae, Araliaceae, Oleaceae, and Amaryllidaceae. Recalcitrant seeds were predicted to be found in 3946 endemic taxa (18%). This percentage was slightly higher in gymnosperms (23%) compared to angiosperms (18%), with dicotyledons showing a higher prevalence than monocotyledons. These taxa spanned 48 families and 436 genera, including 125 gymnosperms and 3821 angiosperms. The percentage of recalcitrant taxa within each family varied substantially, ranging from 0.23% to 100% (Appendix S4). Twenty‐one families had at least 50% of their taxa predicted to bear recalcitrant seeds. Among these, where at least 100 taxa had predicted storage behavior: two families were predominantly recalcitrant (100% of taxa), Lauraceae and Elaeocarpaceae; nine families were more likely recalcitrant (≥ 70%–< 100%) than orthodox, Myristicaceae, Podocarpaceae, Dipterocarpaceae, Fagaceae, Magnoliaceae, Sapotaceae, Arecaceae, Clusiaceae, and Ebenaceae; and two families were marginally recalcitrant (61% to 67%), Sapindaceae and Meliaceae.

**FIGURE 9:**
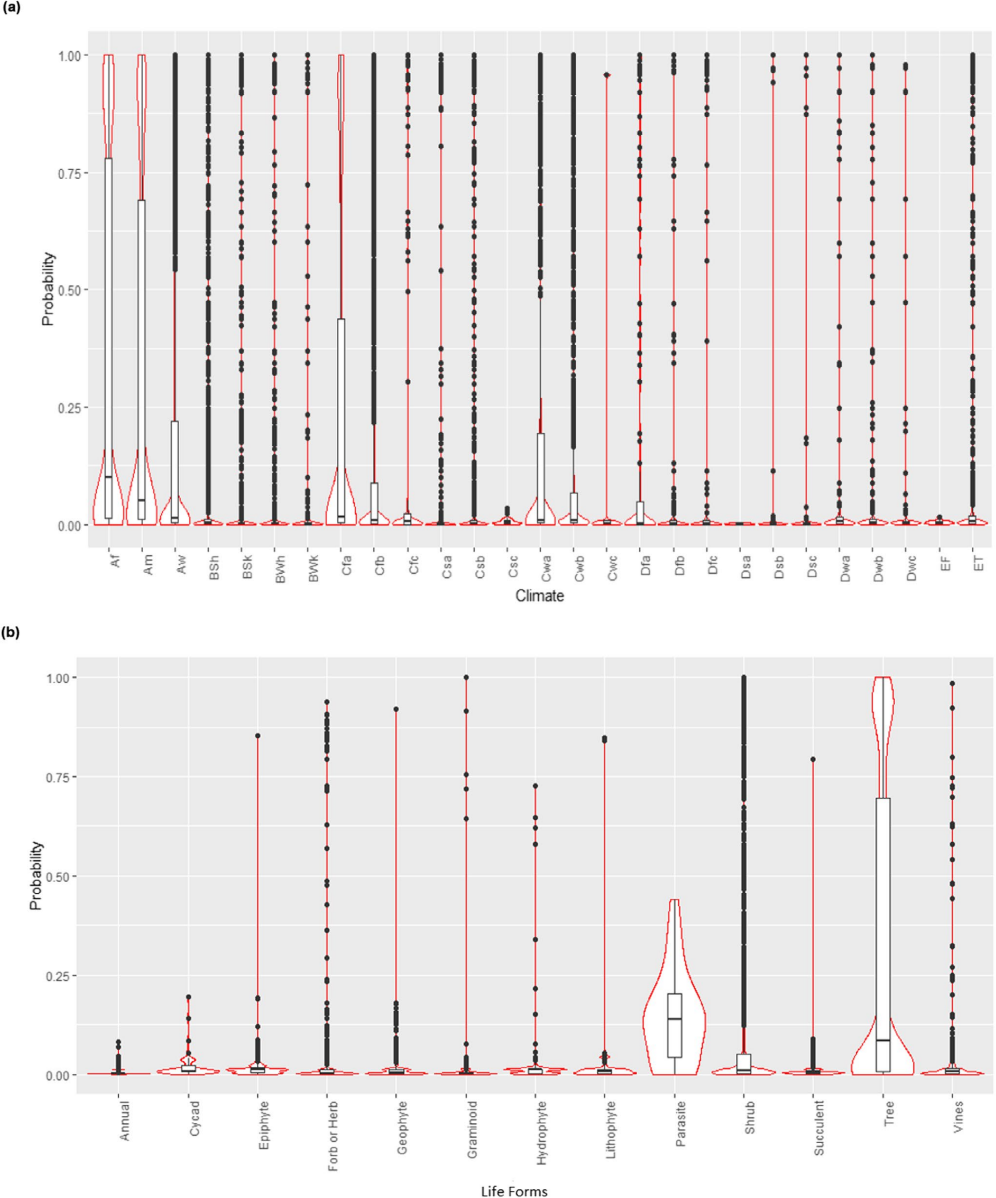
Prevalence of seed storage behavior among endemic vascular plants (*n* = 22,360 taxa): (a) across climate zones; and (b) across life forms. Probabilities closer to zero indicate a higher likelihood of orthodox seed storage behavior, while probabilities closer to one suggest a higher likelihood of recalcitrant seed storage behavior. See Table 4 for climate zone abbreviations.

**FIGURE 10:**
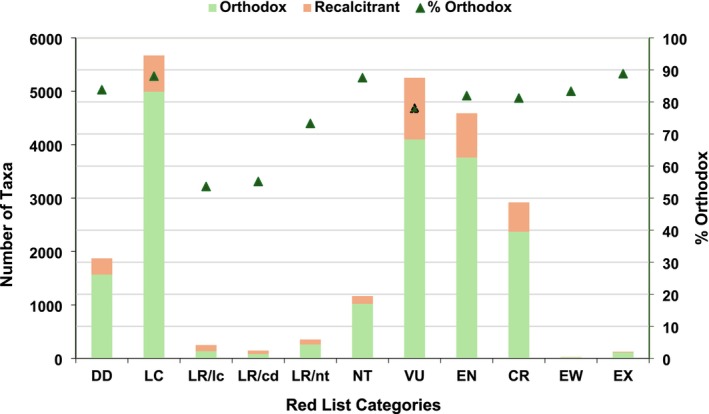
Threatened and non‐threatened endemic vascular plants by seed storage behavior: Orthodox versus recalcitrant (*n* = 22,360 taxa). Global Red List categories follow IUCN (2020).

Recalcitrant seeds were absent in three climate zones, *Csc, Dsa*, and *EF*, all of which had low taxon counts (Figure 9a). In all other zones, recalcitrant taxa were present but consistently less frequent than orthodox. The highest number of recalcitrant taxa occurred in tropical (*Af*t, *Am*) and temperate (*Cfa*) zones. Among 5189 “climate specialists” taxa, about 24% had recalcitrant seeds. Across 13 life forms (Figure 9b), recalcitrant seeds were absent in annuals, cycads, and parasitic plants. In all other life forms, orthodox seeds were more frequent, while recalcitrant seeds were most prevalent in trees, followed by shrubs, and less common among succulents, geophytes, epiphytes, hydrophytes, and lithophytes. From a conservation perspective, about 80% of threatened endemics (including 76% of threatened “climate specialists”) and 85% of non‐threatened endemics can be effectively conserved using conventional seed banking methods (Figure 10).

## 4 Discussion

The IUCN (2020) dataset provides a useful overview of the global distribution of vascular plants, revealing critical insights into endemism, biodiversity, and conservation challenges. Although the IUCN dataset includes nearly 40,000 vascular plant taxa from 250 ISO countries, only a small fraction (0.48%) is cosmopolitan, a pattern that is expected, reflecting both the global rarity of truly widespread taxa and the known biases of the IUCN Red List toward assessing narrowly distributed and threatened taxa. Nonetheless, the IUCN dataset remains one of the most widely used global resources for taxon distribution, and it continues to underpin numerous large‐scale ecological and predictive modeling studies. The occurrence of 23,981 endemic taxa (60% of the total) across 173 ISO countries and diverse families and genera highlights the significance of localized biodiversity, particularly in Madagascar, Ecuador and Australia, which report high levels of endemism. This further highlights the ecological uniqueness of many taxa restricted to specific localities.

Despite the dataset's breadth, significant geographic and taxonomic gaps remain. For example, Europe alone is home to over 20,000 vascular plant species, including around 6200 endemics (Bilz et al. 2011) but the dataset reflects only 2418 native European taxa, with just 910 being endemic. This discrepancy highlights the potential for underrepresentation of key regions, which can skew conservation priorities and resource allocation. Critiques of the IUCN Red List, particularly regarding biases toward specific plant groups, like medicinal plants, specific families, or geographic regions (Bachman et al. 2019), further complicate the issue. These biases can lead to gaps in conservation strategies, as taxa that do not fall into favored categories may be overlooked despite their ecological importance (Glasnović et al. 2024). Given these gaps, the dataset serves as a reliable but partial proxy for understanding global endemic flora. It provides valuable insights into patterns of biodiversity, threats, and conservation needs, but it is crucial to interpret findings with an awareness of the list's limitations.

Mapping endemic occurrences against climates provides a nuanced understanding of how climate influences the distribution of taxon. The high frequency of endemics in tropical and temperate zones, juxtaposed with their scarcity in continental and polar/alpine climates, suggests that climatic stability may foster greater diversification. Thus, the predominance of “climate specialists” in these areas further reinforces the idea that specialized habitats are crucial for supporting endemic biodiversity. Tropical habitats, like rainforests, savannas, mangroves, and coral reefs, are facing unprecedented habitat loss, leading to severe extinction rates among taxa (Bradshaw et al. 2009; Bourgoin et al. 2024). Our findings indicate that 50% to 58% of endemic taxa in the tropics, especially rainforests, are threatened. Climate change, primarily driven by rising temperatures, poses a significant risk to global biodiversity, particularly for taxa with specific habitat requirements and limited distributions. Predicted patterns of spatial distribution for endemic plants under various climate change scenarios suggest that taxa with narrow ecological niches and restricted seed dispersal capabilities may either shift, expand, or narrow their ranges (Banag et al. 2015). In our study, endemics are heavily concentrated in narrow climate ranges, making them “climate specialists”, and 71% of these are threatened, particularly in tropical and temperate climates. This fragility is highlighted by a clear linear trend: the fewer climate zones an endemic occupies, the higher its extinction risk.

Ecological and geographic characteristics of endemic plants across different life forms have been studied to better understand their vulnerability to climate change and land transformation (Broennimann et al. 2006). Climate types can be characterized by examining the relative proportions of each life form in plant communities. The distribution and prevalence of endemic plant life forms in our study provide valuable insights that can inform conservation strategies. Trees constitute the largest group of endemics at 46%, followed by shrubs and forbs/herbs, indicating that woody endemics likely play a critical role in ecosystems. However, the presence of less common life forms suggests that these taxa may also require targeted conservation measures despite their lower numbers. The variation in life form abundance across 27 climate zones emphasizes the importance of habitat specificity. Trees thrive particularly well in certain zones, highlighting their adaptability and ecological significance. Less common or uncommon life forms like graminoids, geophytes, and annuals are more evenly distributed, which may indicate their broader ecological flexibility. The sporadic occurrence of mosses and parasitic plants suggests they may face unique environmental pressures. With 11 life forms having over 50% of their taxa designated as threatened, there is an urgent need to prioritize these groups in conservation planning. Notably, parasitic plants, lithophytes, and mosses are among the most at risk. Moderately threatened life forms, like ferns, epiphytes, and geophytes, may not receive as much conservation attention as trees, yet they further highlight the precarious status of many endemics. Comparatively, annuals, hydrophytes, succulents, and graminoids demonstrate lower threat levels, suggesting that these groups may currently be more resilient to environmental changes. This highlights the susceptibility of certain life forms and the necessity for targeted conservation.

Seed dormancy is a critical factor influencing population dynamics and processes such as colonization, establishment, and adaptation, thereby reducing extinction risk (Willis et al. 2014). However, dormancy can complicate conservation efforts when seeds fail to lose dormancy, thereby limiting their contribution to population viability (Merritt and Dixon 2011). Only 9% of endemic taxa produce exclusively ND seeds, which germinate across a wider range of conditions. ND seeds, which do not enter dormancy after dispersal (Baskin and Baskin 2004a), are common in climates with long growing seasons or among taxa with larger seeds (Rubio de Casas et al. 2017).

PD is the most widespread dormancy (Baskin and Baskin 2004a, 2014), and in our study, it was particularly prevalent among endemics. PD is often linked to specific plant families and habitats, for example, it is common in legumes from tropical environments and widespread among other plant families in temperate habitats (Finch‐Savage and Leubner‐Metzger 2006; Wyse and Dickie 2018). Seeds with non‐deep PD exhibit temperature‐driven physiological transitions from primary dormancy to non‐dormancy or entrance into secondary dormancy (Baskin and Baskin 2004a). Germination of PD seed depends on seasonal variations in temperature, light, precipitation, and, in some cases, oxygen. These seeds often persist in soil seed banks, with longevity shaped by seed traits (e.g., seed size, weight, morphology, etc.), plant traits (e.g., plant height, life form, etc.), ecosystem variables, and burial depth. Transient plants (e.g., annuals) rely on seed dormancy to pass through unfavorable conditions, but prolonged stress can reduce seed viability (Baskin and Baskin 1998). Persistent taxa may retain viable seeds in the soil across multiple germination windows, buffering against environmental variability. In fire‐prone ecosystems, canopy seed banks are an effective survival adaptation; seeds remaining in closed fruits or cones for years, sometimes over a decade, and release in response to fire, after which the seeds have a short lifespan (Enright et al. 2002). Another strategy to avoid unfavorable conditions is long‐distance dispersal, dependent on plant and seed traits like size, weight, morphology, life form, and dispersal mechanisms (wind, water, animals). However, many endemics exhibit short‐distance seed dispersal due to limited adaptations (Xu et al. 2018). For instance, in southern Iberia, 45% of endemic plants lack effective seed dispersal mechanisms (Giménez et al. 2004), preventing their seeds from reaching suitable habitats for germination.

PY, found in 8% of our taxa, occurs in 15 angiosperm families (Baskin et al. 2008). PY is broken by environmental triggers like temperature fluctuations, fire, drying, freezing/thawing, or animal digestion (Baskin and Baskin 1998). Once broken, seeds cannot re‐enter dormancy, making them more sensitive to climate‐induced change of environmental conditions than PD seeds (Baskin et al. 2008). While seed coats deter predation (Paulsen et al. 2013), PY is linked to higher extinction rates and negative diversification, whereas PD and possibly MD are associated with increased diversification (Baskin and Baskin 2014).

MD is rare, found in only 1% of endemics in our study. MD seeds require time for embryo growth post‐dispersal but do not rely on external environmental cues for dormancy breaking (Baskin and Baskin 2004a). In contrast, MPD seeds, present in 11% of endemic taxa, require both internal embryo growth and specific environmental cues (e.g., temperature stratification) for germination (radicle emergence).

DUST seeds, which are extremely small and lack endosperm, have evolved independently in at least 12 plant families, including Orchidaceae (Prasongsom et al. 2022). We identified 17 endemic taxa across four families (Balanophoraceae, Burmanniaceae, Rafflesiaceae and Triuridaceae) with DUST seeds (eight parasitic plants, eight forbs/herbs, and one epiphyte). These taxa often exhibit MD or MPD traits and may have unique ecological and germination requirements (Leake 1994; Baskin and Baskin 2004b; Eriksson and Kainulainen 2011). All 906 Orchidaceae taxa in our list were identified as having MPD, although their extremely small seed size also qualifies them as possessing DUST seeds. ND has been identified in some *Dendrobium* species due to the presence of relatively large embryos *cf* the seed volume (Prasongsom et al. 2022).

About 92% of world flora are predicted to produce desiccation‐tolerant seeds, with taxa sharing similar ecological and physiological traits often exhibiting comparable seed storage behaviors. Our findings indicate that 82% of endemic taxa may possess orthodox seeds and are suitable for conventional seed banking methods, aligning closely with New Zealand's indigenous flora (83%; Wyse et al. 2023), though 10% below the global average. However, since intermediate seed storage behavior was not distinguished in our analysis, some taxa predicted to be orthodox may in fact fall into this category, limiting their long‐term seed conservation potential. For instance, although some orchids produce relatively long‐lived, storable seeds (Francisqueti et al. 2024), many are short‐lived and may fall into the intermediate class.

Approximately 18% of endemic taxa in our dataset, 10% above the global average, are predicted to have recalcitrant seeds, including 24% of “climate specialists”. These were concentrated in tropical (*Af*, *Am*) and temperate zone (*Cfa*), which also support high endemic richness. Recalcitrant seeds are most prevalent in tropical regions, particularly among phanerophytes and in moist, relatively seasonal climates, with the highest occurrence in non‐pioneer, evergreen rain forest trees (Tweddle et al. 2003; Wyse et al. 2017, 2018a; Subbiah et al. 2019). Their frequency decreases in arid and highly seasonal or cooler environments. According to Tweddle et al. (2003), among shrubs and trees, desiccation sensitivity was more frequent in ND than dormant seeds, and in PD (~12%) than PY (1%) and PYPD (0%) seeds. In our study, it was more common in trees, followed by shrubs while absent among annuals, cycads, and parasitic plants.

Many threatened plants, particularly from moist rain forests, produce recalcitrant seeds and are unsuitable for conventional seed banking (Wyse et al. 2018b). This highlights the need for alternative ex situ measures such as cryopreservation of tissues (often seed embryonic axes and embryogenic cells), which is increasingly recommended as a viable alternative for preserving their genetic diversity (Li and Pritchard 2009; Hay and Probert 2013; Ballesteros and Pritchard 2020; Walters and Pence 2020; Liu et al. 2024).

Overall, our findings highlight the importance of a multifaceted approach to developing conservation strategies that incorporate a taxon's climate relationships and trait‐based resilience. Adaptive conservation measures should account for: (i) climate variability and distributional shifts, with a priority on “climate specialists” and taxa with narrow climate ranges, particularly in high‐risk regions; (ii) the ecological significance, diversity, and complexity of functional trait variation, especially endemic taxa representing susceptible life forms or those producing dormant or recalcitrant seeds; and (iii) enhancing seed banking practices, investing in cryobiotechnology, and advancing research on seed storage and propagation to support effective long‐term conservation.

## Author Contributions

**Udayangani Liu:** conceptualization (lead), data curation (equal), formal analysis (lead), investigation (lead), methodology (lead), writing – original draft (lead), writing – review and editing (lead). **Tiziana Antonella Cossu:** data curation (equal), formal analysis (supporting), methodology (supporting), software (lead), visualization (lead), writing – original draft (supporting), writing – review and editing (supporting). **Hugh W. Pritchard:** conceptualization (supporting), investigation (supporting), methodology (supporting), supervision (lead), writing – original draft (supporting), writing – review and editing (supporting).

## Conflicts of Interest

The authors declare no conflicts of interest.

## Supporting information


**Appendix S1:** ece371881‐sup‐0001‐Appendix.xlsx.

## Data Availability

The primary data supporting the findings of this study are publicly available from the IUCN Red List of Threatened Species (https://www.iucnredlist.org/), the Global Biodiversity Information Facility (https://www.gbif.org/) and literature (Baskin and Baskin 2014; Willis et al. 2014).
